# Large Jejunal Gallstone Ileus After Sleeve Gastrectomy

**DOI:** 10.7759/cureus.90148

**Published:** 2025-08-15

**Authors:** Michael C Wilkinson, Esther Wu

**Affiliations:** 1 Department of Surgery, Loma Linda University Health, Loma Linda, USA

**Keywords:** bariatric surgery, enterolithotomy, gallstone ileus, intestinal obstruction, sleeve gastrectomy

## Abstract

Gallstone ileus is a rare but serious complication in patients with cholelithiasis, resulting in an increased risk of mortality, highlighting the importance of timely diagnosis and management. Excess weight loss after bariatric surgery is associated with more pronounced symptomatic gallstone disease. We present a 44-year-old female with a previous vertical sleeve gastrectomy who presented with progressively worsening mechanical small bowel obstruction. Computed tomography showed cholelithiasis, and magnetic resonance cholangiopancreatography identified extravasation of contrast from the gallbladder to the duodenum. Laparotomy with enterolithotomy revealed a large gallstone completely obstructing the proximal jejunum. In patients with prior bariatric surgery presenting with symptoms of small bowel obstruction, the diagnosis of gallstone ileus requires a high index of suspicion. While the optimal procedure is heavily debated, one-stage procedures should be reserved for patients who are clinically optimized. Gallstone ileus is a rare but serious diagnosis not isolated to the comorbid elderly. Further studies are needed to assess the incidence of gallstone ileus after bariatric surgery.

## Introduction

Gallstone ileus presents as a rare but serious complication for patients with cholelithiasis (0.4%) and is the underlying etiology in 1-4% of patients with mechanical small bowel obstruction [1-2]. While more common in the elderly (70-80s), younger patients have been reported [3]. Gallstone ileus is more common in women with a F: M predominance of 4: 1 [4-5]. Studies have reported an estimated mortality between 8% and 15% due to both a delay in diagnosis and other concomitant conditions. In patients who have undergone previous weight-reduction surgery, symptomatic gallstone disease is more pronounced in the first 5 years, with excess risk coinciding with the most weight loss in the first 2 years [6-7]. To our knowledge, very few cases of proximal gallstone ileus have been reported in the literature, and there are no studies that have evaluated the incidence of gallstone ileus after bariatric surgery, nor are there any prior reports of gallstone ileus after vertical sleeve gastrectomy [8]. In this case report, we aim to increase awareness of the diagnosis and management of gallstone ileus after bariatric surgery.

## Case presentation

**Figure 1:**
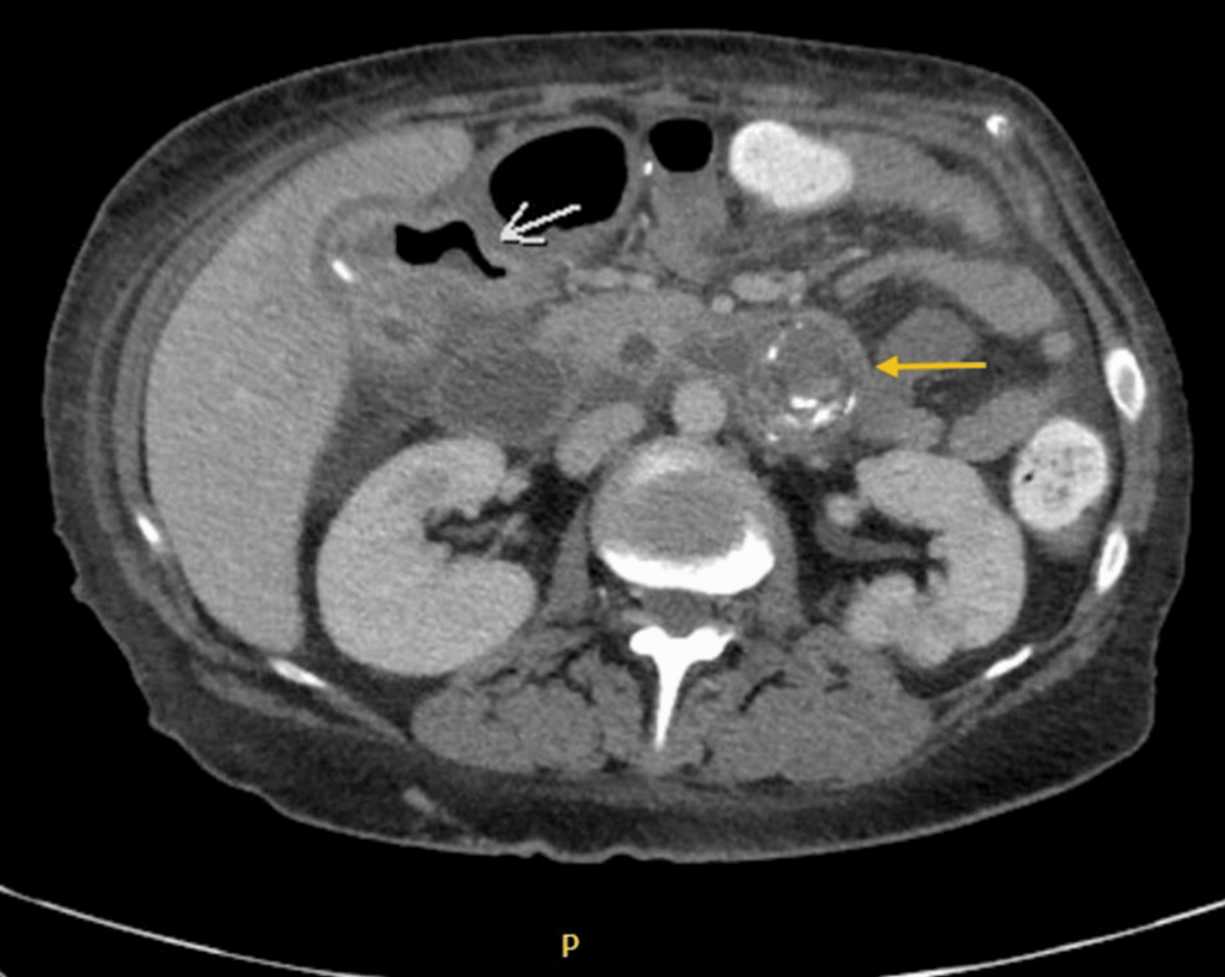
Contrast-enhanced axial CT image of cholecystoduodenal fistula (white arrow) and large jejunal gallstone (gold arrow).

We present a 44-year-old female with a past medical history of obesity with a previous vertical sleeve gastrectomy two years prior, who presented to our institution with abdominal pain, oral intolerance, nausea, bilious non-bloody emesis, and constipation for 14 days prior to transfer from another hospital, where she was treated conservatively with intravenous fluids and antibiotics. The patient was initially evaluated in the outpatient setting by her bariatric surgeon, who sent her to urgent care to obtain a computed tomography (CT) scan, which demonstrated cholelithiasis, pneumobilia, and a dilated common bile duct (CBD) to 9 mm (Figure 1).

**Figure 2:**
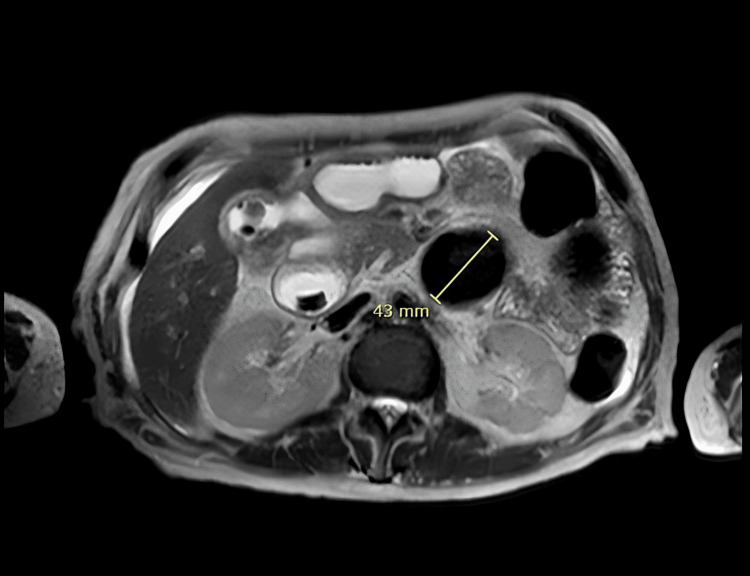
T2-weighted magnetic resonance axial image of a large gallstone, cholelithiasis, and cholecystoduodenal fistula.

The patient was admitted to a local hospital, where a magnetic resonance cholangiopancreatography (MRCP) was obtained, which showed extravasation of contrast from the gallbladder to the duodenum, suggestive of a cholecystoduodenal fistula. The patient was subsequently transferred to our institution for higher-level care (Figure 2). 

Upon arrival, the patient endorsed a known history of gallstones since her sleeve gastrectomy, with prior ultrasound imaging demonstrating cholelithiasis. Her abdomen was mildly distended but soft, with audible bowel sounds. Blood tests revealed no leukocytosis, elevated liver function tests, or hyperbilirubinemia. She was moderately malnourished. A Gastrograffin (Bayer: Leverkusen, Germany) small bowel follow-through demonstrated poor transit time with findings suggestive of partial obstruction. Initial management included conservative intravenous fluids, broad-spectrum antibiotics, and parenteral nutrition.

**Figure 3:**
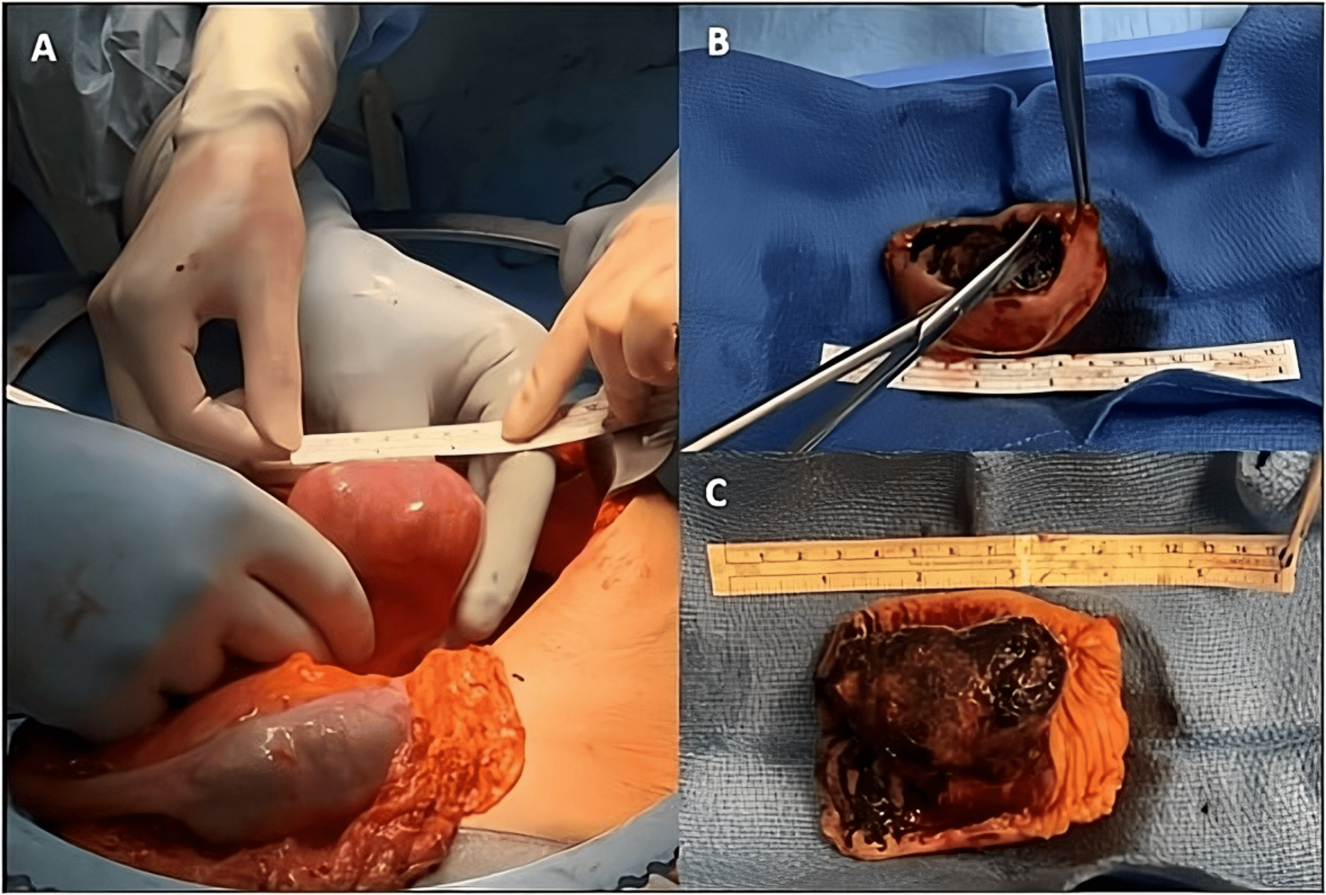
Intraoperative finding of gallstone ileus in the proximal jejunum (A). Jejunal resection specimen (B). Large jejunal gallstone in preserved orientation measuring 7 cm × 5 cm × 4 cm (C).

On hospital day 2, the patient became more distended with worsening abdominal pain, nausea, and bilious emesis, including a subcentimeter gallstone, prompting nasogastric decompression. On hospital day 3, the patient underwent an urgent exploratory laparotomy for worsening distension. Intraoperative findings demonstrated a large gallstone impacting the proximal jejunum 50 cm distal to the Ligament of Treitz. The stone was impacted despite attempts to milk it proximally; therefore, a segmental small bowel resection was performed with creation of a primary side-to-side antiperistaltic stapled anastomosis. The specimen was opened on the back table and measured 7 cm × 5 cm × 4 cm (Figure 3). The patient had an unremarkable postoperative course, tolerated a regular diet, and pain was well-controlled. She was discharged home on postoperative day 3 with close outpatient follow-up, where she recovered and subsequently underwent an elective successful open cholecystectomy, resection of choledochoduodenal fistula with tangential partial duodenectomy, and primary stapled repair of the duodenum for persistent postprandial abdominal pain.

## Discussion

Cholelithiasis after bariatric surgery is not uncommon, with rates of asymptomatic and symptomatic gallstone disease after ultrasound surveillance within a year from surgery ranging from 30-53% and 7-16%, respectively [9-11]. The pathophysiology of stone formation after bariatric surgery has been attributed to rapid weight loss. An increased presence of prostaglandins and arachidonic acid in bile, along with increased mobilization of cholesterol from tissue stores, is excreted in bile, leading to stone formation [10,12]. Rates of symptomatic stone formation have been reportedly higher after bypass surgery compared to sleeve gastrectomy due to decreased cholecystokinin-mediated gallbladder contraction, duodenal exclusion, and denervation of the hepatic branch of the left vagus nerve. However, a large retrospective review analyzing rates of symptomatic gallstone disease after bariatric surgery found no difference between gastric bypass and sleeve gastrectomy (8.7% vs 3.8%, p = 0.296) [13].

Gallstones enter the intestines through a bilioenteral fistula, usually after an episode of acute cholecystitis. Local pressure exerted by the gallstone in combination with surrounding inflammation facilitates the development of a fistulous tract. Gallstones less than 2.5 cm usually pass spontaneously while stones great than 2.5 cm can cause obstruction, mostly notably in the distal ileum and ileocecal valve due to a smaller luminal diameter and reduced peristaltic activity (distal ileum 60-65%, jejunum 16.1%, stomach 14.2%, colon 0.5-4.1%, sigmoid 2-8%, duodenum 3.5-10%) [4,8,14]. Obstructing stones range from 0.5 to 6 cm, while the largest stone reported was 17.7 cm by Turner et al [15]. When gallstones obstruct the proximal small bowel, leading to gastric outlet obstruction, this is known as Bouveret syndrome.

The morbidity and mortality of gallstone ileus remain high, necessitating timely diagnosis. Classically, patients present with nonspecific nausea, vomiting, persistent or recurrent episodes of abdominal distention and colicky pain, and diarrhea, but rarely jaundice. A high index of suspicion, in addition to proper diagnostic imaging, aids in making a timely diagnosis [5]. Upright abdominal radiographs are useful for demonstrating the classic Rigler’s Triad (multiple air fluid levels demonstrating small bowel obstruction, pneumobilia, and an ectopic gallstone); however, this has only been found in 40-50% of cases [14,16]. In one report, air in the gallbladder was also a frequent finding in gallstone ileus [17]. CT remains the gold standard for identifying stone and fistula location as well as surgical planning, with an overall sensitivity, specificity, and accuracy of 93%, 100%, and 99%, respectively [18].

The appropriate surgical management for gallstone ileus remains without a uniform consensus. Elderly patients with electrolyte abnormalities and other medical comorbidities should be managed with initial enterolithotomy alone. For younger, more fit, and medically optimized patients, a definitive one-stage enterolithotomy, cholecystectomy, and repair of fistula have been shown to have lower rates of stone recurrence, gallbladder malignancy, fistulous bleeding, fat malabsorption, and cholangitis [1,19,20]. However, in the largest review of the one-stage procedure, which included 1001 cases, mortality was 16.9% compared to 11.7% for simple enterolithotomy alone [4]. Moreover, recurrent gallstone ileus is less than 5% and definitive cholecystectomy may not be required for high surgical risk patients with large draining fistulous tracts. The presence of choledocholithiasis or biliary colic with or without postprandial abdominal pain is an indication to proceed with cholecystectomy [3].

## Conclusions

Gallstone ileus is a rare but serious disease that affects younger patients after bariatric surgery. As bariatric surgery is associated with more profound symptomatic cholelithiasis, a high index of suspicion for gallstone ileus is warranted in patients presenting with small bowel obstruction. CT remains the gold standard for early and accurate diagnosis, and enterolithotomy alone may be an appropriate surgical strategy depending on patient age, comorbidities, and performance status. Additional studies are needed to investigate the phenomenon of gallstone ileus after weight-loss surgery.
